# Testosterone modulates cardiac contraction and calcium homeostasis: cellular and molecular mechanisms

**DOI:** 10.1186/s13293-015-0027-9

**Published:** 2015-04-29

**Authors:** Omar Ayaz, Susan Ellen Howlett

**Affiliations:** Department of Pharmacology, Dalhousie University, 5850 College Street, Sir Charles Tupper Medical Building, PO Box 15000, Halifax, NS B3H 4R2 Canada; Medicine (Geriatric Medicine), Dalhousie University, 5850 College Street, PO Box 15000, Halifax, NS B3H 4R2 Canada

**Keywords:** Aging, Excitation-contraction coupling, Gonadectomy, Orchiectomy

## Abstract

The incidence of cardiovascular disease rises dramatically with age in both men and women. Because a woman’s risk of cardiovascular disease rises markedly after the onset of menopause, there has been growing interest in the effect of estrogen on the heart and its role in the pathophysiology of these diseases. Much less attention has been paid to the impact of testosterone on the heart, even though the levels of testosterone also decline with age and low-testosterone levels are linked to the development of cardiovascular diseases. The knowledge that receptors for all major sex steroid hormones, including testosterone, are present on individual cardiomyocytes suggests that these hormones may influence the heart at the cellular level. Indeed, it is well established that there are male-female differences in intracellular Ca^2+^ release and contraction in isolated ventricular myocytes. Growing evidence suggests that these differences arise from effects of sex steroid hormones on processes involved in intracellular Ca^2+^ homeostasis. This review considers how myocardial contractile function is modified by testosterone, with a focus on the impact of testosterone on processes that regulate Ca^2+^ handling at the level of the ventricular myocyte. The idea that testosterone regulates Ca^2+^ handling in the heart is important, as Ca^2+^ dysregulation plays a key role in the pathogenesis of a variety of different cardiovascular diseases. A better understanding of sex hormone regulation of myocardial Ca^2+^ homeostasis may reveal new targets for the treatment of cardiovascular diseases in all older adults.

## Review

### Introduction

Cardiovascular diseases are a leading cause of hospitalization and death for both men and women [1]. As advanced age is a major risk factor for the development of cardiovascular disease in both sexes, the incidence and prevalence of these diseases is expected to escalate as our population ages [2]. The knowledge that the incidence of cardiovascular disease in women rises as estrogen levels fall after menopause has fueled interest in its potential cardioprotective effects [3]. What is less well appreciated is that testosterone levels also decline with advancing age, not just in men but in women too [4-6]. This suggests that low-testosterone levels may contribute to the pathogenesis of cardiovascular disease. Indeed, a number of clinical studies have shown that low endogenous levels of testosterone are associated with cardiovascular disease [4]. Furthermore, testosterone-replacement therapy, which is used to treat testosterone deficiency secondary to aging [7], may have beneficial effects in the setting of heart failure and ischemic heart disease [4,8,9].

Despite the popularity of testosterone supplementation in older adults, how testosterone affects the heart is not fully understood. The discovery of androgen receptors in individual heart cells (myocytes) [10,11] suggests that testosterone might modulate heart function, at least in part, by effects on the ventricular myocytes themselves. Here, we review emerging evidence that suggests testosterone influences myocardial function at the cellular level by modifying processes involved in intracellular Ca^2+^ homeostasis. As disruption of Ca^2+^ handling plays a key role in many cardiovascular diseases [12,13], understanding the mechanisms underlying the effects of testosterone on myocardial Ca^2+^ homeostasis may help explain its influence on cardiovascular health.

### Testosterone

The principal male sex hormone, testosterone, is an androgen steroid. Testosterone plays important roles in normal growth and development, and its levels decline with age in both men and women. The following discussion provides a broad overview of testosterone, and its receptors, including pathways involved in its biosynthesis, regulation, and metabolism.

#### Testosterone in men and women

Testosterone is produced primarily by the testes in men, although it also can be produced by the adrenal glands and other sites including adipose tissue and bone [14,15]. It is responsible for testes descent and reproductive tract development in the fetus, development of male secondary sex characteristics in puberty, and the production of sperm [16,17]. Testosterone production in men begins *in utero*, rises sharply in puberty, and then declines with age [17,18]. Indeed, the Massachusetts Male Aging Study showed that total serum testosterone levels decline by 1.6% per year starting at age 40 [19]. Testosterone is also produced by the ovaries, the adrenal glands, and tissues such as adipose tissue and skin in women [6,15,20,21], although serum concentrations are almost 20-fold lower in pre-menopausal women compared to age-matched men [22]. Interestingly, testosterone levels also decline with age in women [6,23]. This age-dependent reduction in testosterone is not restricted to humans, as it is also seen in older male rats and mice (>20 months of age) in conjunction with a decline in fertility [24,25], although whether levels decline in aged female animals has not been investigated.

#### Biosynthesis of testosterone

**Figure 1:**
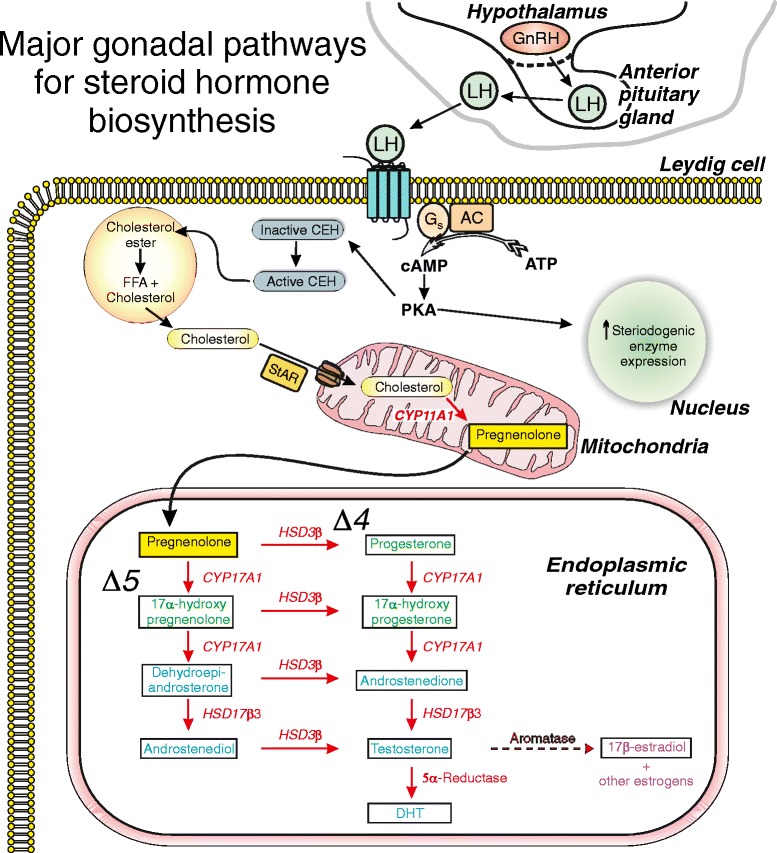
Major gonadal pathways for testosterone biosynthesis. Gonadotropin-releasing hormone (GnRH) secreted from the hypothalamus releases luteinizing hormone (LH) from the pituitary. LH binds to LH receptors on Leydig cells, stimulates G_s_, and activates the cAMP/protein kinase A (PKA) pathway. PKA promotes the transport of cholesterol into mitochondria and increases transcription of genes involved in testosterone biosynthesis. Cholesterol is converted to pregnenolone, which diffuses into the endoplasmic reticulum for testosterone biosynthesis via ∆^4^ and ∆^5^ pathways. Testosterone is formed by 17β-hydroxysteroid dehydrogenase 3 (HSD17β3) in the ∆^4^ pathway and by 3β-hydroxysteroid dehydrogenase (HSD3β) in the ∆^5^ pathway. Testosterone is converted to dihydrotestosterone (DHT) by 5α-reductase, and some are aromatized to 17ß-estradiol.

Testosterone biosynthesis in men is controlled by the hypothalamic-pituitary-gonadal axis [26], as shown in the overview of major gonadal pathways for testosterone biosynthesis in Figure 1. The first step is the release of gonadotropin-releasing hormone (GnRH), which is synthesized and secreted from hypothalamic neurons [27]. GnRH binds to receptors on the anterior pituitary gland and stimulates the synthesis and secretion of luteinizing hormone (LH) into circulation [26,28]. LH binds to LH receptors on Leydig cells in the testes and stimulates a G-protein, G_s_, to activate the cAMP/protein kinase A (PKA) pathway. This promotes the transport of cholesterol into the mitochondria and increases transcriptional activation of gene-encoding enzymes involved in testosterone biosynthesis [29]. The levels of testosterone in circulation are under tight hormonal regulation via a negative feedback mechanism that prevents the release of GnRH and LH when testosterone levels are high [26].

When serum testosterone levels are low, cholesterol in Leydig cells is transported to the inner mitochondrial membrane via a multi-protein complex in conjunction with the steroidogenic acute regulatory (StAR) protein [30], as shown in Figure 1. The rate-limiting reaction in the production of steroid hormones is the conversion of cholesterol to pregnenolone by the cholesterol side chain cleavage enzyme, a cytochrome P450 (CYP) enzyme known as CYP11A1 [30]. When pregnenolone is formed, it diffuses into the endoplasmic reticulum where testosterone biosynthesis proceeds via ∆^4^ and ∆^5^ pathways (Figure 1). In the ∆^4^ pathway, pregnenolone is converted to progesterone by 3β-hydroxysteroid dehydrogenase (HSD3β) [15,31,32]. CYP17A1 converts progesterone to 17α-hydroxyprogesterone by a hydroxylation reaction and then to androstenedione by a lysis reaction. Testosterone is formed by 17β-hydroxysteroid dehydrogenase 3 (HSD17β3). In the ∆^5^ pathway, CYP17A1 converts pregnenolone (via a hydroxylation reaction) to 17α-hydroxypregnenolone followed by a lysis reaction to yield dehydroepiandrosterone (DHEA) [15,31,32]. DHEA is then converted to androstenedione by HSD3β. In the final step, HSD17β3 converts androstenedione to testosterone. DHEA can also be converted to androstenediol by HSD17β3 and then converted to testosterone by HSD3β [15,31]. Some testosterone is converted to the more potent androgen, dihydrotestosterone (DHT), by 5α-reductase [31]. In the Leydig cells, the major androgens (testosterone and DHT) leave by passive transport into circulation, where most bind to proteins including sex-hormone-binding globulin and/or albumin, although some circulate as free androgen [26].

Figure 1 also demonstrates that the enzyme aromatase can convert testosterone to the primary estrogen, 17ß-estradiol. This is a minor pathway in the Leydig cell. Interestingly, aromatase also is present in a number of extragonadal sites including adipose tissue, bone, and the brain in both men and women [33]. Furthermore, cardiac-specific expression of aromatase has recently been shown in the adult mouse heart [34]. Conversion of circulating testosterone to 17ß-estradiol by these tissue-based aromatase pathways may increase 17ß-estradiol levels under conditions such as obesity [35]. Furthermore, certain anabolic steroids, including testosterone esters as well as nortestosterone derivatives (e.g., nandrolone decanoate and nandrolene phenpropionate), can be aromatized to estradiol [36]. Indeed, some anabolic steroid users take anti-estrogens to minimize adverse effects (e.g., gynecomastia) associated with the aromatization of testosterone derivatives to estradiol [37]. Thus, effects of testosterone supplementation with derivatives that can be aromatized may actually be due, at least in part, to estradiol rather than androgen. As a consequence, some studies of the effect of androgens on the cardiovascular system use the non-aromatizable androgen DHT, rather than testosterone or its derivatives, for example [38-40].

As discussed in the ‘Testosterone in men and women’ section, there is evidence that circulating testosterone decreases with age in both men and women [6,18,19,23]. In aging men, the fall in serum testosterone is largely due to a decrease in the ability of Leydig cells to produce testosterone in response to LH [41]. This arises as a result of age-associated attenuation of the cAMP/PKA pathway, leading to less transfer of cholesterol into the mitochondria and a reduction in the production of steroidogenic enzymes [41]. Interestingly, a similar mechanism has been proposed to lead to the age-dependent decrease in testosterone levels in male rats [42]. In women, the age-dependent decline in circulating testosterone is thought to result from a combination of events including ovarian failure plus a reduction in the adrenal production of androgens [6]. Thus, the aging process reduces the amount of testosterone available to interact with androgen receptors, as described in the next section.

#### Androgen receptors in the heart

Biological effects of androgens are mediated by interactions with androgen receptors. The effects of androgens on androgen receptors associated with the male reproductive system are well documented [43]. The knowledge that these receptors are present in tissues such as the liver, kidney, brain, skeletal muscle, and heart [10,44,45] has fueled interest in the role of androgens in regulation of physiological processes including myocardial function. Classic receptor-binding studies provided the first evidence for androgen receptors in the atria and ventricles of the heart [46,47]. Other studies showed that mRNA for the androgen receptor is present in cardiomyocytes isolated from men and women, as well as in cardiomyocytes from rats and dogs [10]. More recent work has shown that androgen receptor protein is expressed in both the atria and ventricles of male and female mice [11]. This latter study also shows that androgen receptors are predominantly expressed in the cytosol and nucleus of heart tissue from adult mice [11]. Together, these studies demonstrate that androgen receptors are present in the heart and raise questions about the role of testosterone in the regulation of myocardial function.

Most of the biological actions of endogenous and exogenous androgens are genomic effects mediated by androgen receptors that are members of the nuclear receptor gene superfamily. In the absence of androgens, the androgen receptor forms a complex with heat-shock proteins in the cytosol [43]. The binding of androgens causes receptor dissociation from heat-shock proteins and translocation to the nucleus, where the ligand-activated receptor homodimerizes and associates with chromatin by DNA-binding or through binding to other chromosomal proteins [43]. This causes transcriptional activation or repression of androgen-responsive genes, which are cell-specific according to transcription factors and cis-acting DNA elements in the particular tissue [48,49]. Transcriptional activation of androgen-responsive genes results in slow, long-lasting effects that can persist for hours after androgen receptor stimulation [50]. This androgen-mediated transcriptional regulation is considered the canonical/genomic pathway for testosterone signaling and is thought to be responsible for most of the effects of androgens on the heart, as discussed in the ‘Effects of testosterone on cardiac contractile function’ section below.

While the classic genomic pathway mediates many of the biological effects of androgens, it is unlikely to be responsible for the rapid responses to androgens observed in some tissues, including the cardiovascular system. These transcription-independent effects are thought to be mediated by the activation of a nongenomic pathway, although the underlying molecular mechanisms are not well understood [43]. For example, there is strong evidence that testosterone induces rapid vasorelaxation in both large arteries and smaller resistance vessels [51]. Acute application of androgens also increases intracellular Ca^2+^ levels in osteoblasts, platelets, skeletal muscle cells, neurons, and, importantly, in cardiac myocytes [52]. Indeed, testosterone rapidly elicits voltage-dependent Ca^2+^ oscillations and IP_3_-receptor-mediated Ca^2+^ release from internal stores in neonatal rat cardiomyocytes [53]. While a cell-membrane-associated androgen receptor may be responsible, at present, its identity is unknown and other mechanisms, such as direct activation of ion channels and signaling pathways, may be involved [43,51,52]. Thus, although the canonical/genomic pathway is primarily responsible for the effects of androgens on the heart, the nongenomic pathway also may contribute.

### Effects of testosterone on cardiac contractile function

#### Influence of androgens on myocardial contractility in humans

Clinical studies have shown that there are male-female differences in the ability of the heart to contract, even in the absence of cardiovascular disease. For example, women have a higher ejection fraction at rest than men [54], but men respond to exercise with a greater increase in ejection fraction than women [55,56]. There is growing experimental evidence that estrogen plays a role in these male-female differences in myocardial contractility [57], although testosterone also may contribute.

The influence of androgens on myocardial contractility in humans has been investigated by examining the effects of anabolic-androgenic steroids on heart function. These steroids are synthetic derivatives of testosterone that are used therapeutically, in particular, to stimulate muscle growth [58]. They also are used as performance-enhancing drugs in sports, where their use is banned due to potential unfair advantage as well as adverse effects of high doses of these drugs [59]. While some echocardiography studies report that left ventricular mass is increased by anabolic steroid use [60-63], others report no effect [64-67], and the apparent increase in mass is abolished when it is indexed to fat-free body mass [68]. Echocardiography studies also have shown that anabolic steroid use adversely affects myocardial function, although again the data are not consistent. While some report that ejection fraction is attenuated by chronic anabolic steroid use [60,68], others have found no change in ejection fraction in steroid users [61,63-66]. Similarly, although some studies report that anabolic steroid use slows cardiac relaxation [60-63,68], others report no effect [64-66]. These divergent results likely reflect the difficulties inherent in studies of anabolic steroid use. These include differences in drugs between and within studies, variations in the doses used and the difficulty in obtaining a precise history of drug use in participants. Nonetheless, these clinical studies provide evidence that testosterone may influence cardiac contractile function. This has been explored in more detail in pre-clinical models, as outlined below.

#### The impact of testosterone on cardiac contractile function in animal models

Various animal models have been used to investigate the influence of testosterone on myocardial function *in vivo* and in intact hearts. Some investigators have examined the influence of long-term supplementation with testosterone, or other androgens, on cardiac contractile function. However, most have inferred information about chronic testosterone effects from gonadectomy (GDX) experiments, where animals were subjected to bilateral removal of the testes (±testosterone replacement) for varying periods of time. As discussed in detail below, results of these investigations demonstrate that male sex hormones modulate cardiac contractile function in animal models.

Relatively little is known about the influence of testosterone on cardiac contractile function in animal models *in vivo*, although one study has investigated the effect of GDX on myocardial structure and function with M-mode echocardiography [69]. These authors report that 10 weeks of hormone deprivation attenuates contractile function, as demonstrated by a decrease in both fractional shortening and ejection fraction compared to hormone-replete control mice [69]. GDX also causes concentric remodeling of the heart, characterized by increased left ventricular posterior wall and reduced left ventricular internal diameter in diastole, as well as increased relative wall thickness when compared to intact males [69]. These echocardiographic data suggest that prolonged absence of male sex hormones modifies the structure and function of the heart. Still, whether this affects cardiac relaxation and promotes diastolic dysfunction has not yet been investigated with techniques such as tissue Doppler. Such studies would be of considerable interest, as there is evidence that GDX slows relaxation in intact hearts and in isolated myocytes, as discussed below.

A number of studies have explored the effects of testosterone supplementation on myocardial contractility in intact hearts isolated from various animal models. These studies typically used various anabolic-androgenic steroids at high doses to mimic anabolic steroid abuse in exercise training. Most found that chronic administration (8–12 weeks) of 5–50 mg/kg/week of anabolic steroid (e.g., nandrolone decanoate, stanozolol, or 17α-methyltestosterone) suppresses peak left ventricular contractile performance in young adult rats ([70-72] but *c.f.* [73]). By contrast, high doses of anabolic steroids have no effect on the rates of left ventricular pressure rise (+dP/dT) or the rates of left ventricular pressure decay (−dP/dT), which indicates that these agents do not affect the time course of contraction or relaxation ([70,71,73] but *c.f.* [72]). The impact of more physiological steroid concentrations also has been examined. Eleawa et al. [74] treated young intact rats with concentrations of testosterone propionate (1.5 mg/kg/week; 12 weeks) designed to produce plasma testosterone levels between 3–11 ng/ml, to mimic more physiological concentrations. They found that these lower levels of testosterone had no effect on left ventricular developed pressure (LVDP), +dP/dT, or − dP/dT in Langendorff-perfused hearts [74]. These observations indicate that while high levels of anabolic-androgenic steroids can negatively affect peak cardiac contractile performance, lower concentrations have few effects in animal models.

Other investigators have examined the influence of chronic testosterone withdrawal on cardiac contractility in GDX rodents, as summarized in Table 1. Studies in Langendorff-perfused hearts show that LVDP is not affected by short-term (2–9 weeks) GDX [75,76], but declines after longer-term GDX (e.g., 16 weeks) [74]. There is evidence that these deficits in contractile function in low-testosterone states may be more prominent at high physiological loads (Table 1). For example, LVDP is attenuated 3–10 weeks after GDX in working hearts models, but only when hearts are exposed to high left atrial pressures [77,78]. Furthermore, although left ventricular end-diastolic pressure (LVEDP) is unaffected by GDX in Langendorff-perfused [75] and catheterized hearts [69], it declines when GDX hearts are subjected to high left atrial pressures [77,78]. The time course of contraction is also modified by withdrawal of male hormones (Table 1). While most studies report that + dP/dT is not affected by GDX ([69,76] but *c.f.* [74]), there is evidence that -dP/dT is slowed following GDX ([74,77,78] but *c.f.* [69,76]). Consistent with these results, recent work has shown that + dP/dT is also slower in young adult male mice that overexpress aromatase and have much lower testosterone levels (and higher estrogen levels) compared to wild type controls [79]. Interestingly, these changes in the amplitude and time course of cardiac contraction in hearts from GDX animals are reversed by testosterone replacement [74]. Together, these observations suggest that chronic testosterone withdrawal attenuates peak contraction and slows relaxation, especially when hearts are working under high loads. As cardiac contraction is initiated by a transient rise in cytosolic Ca^2+^ in individual cardiomyocytes, effects of testosterone on contractile function may arise from effects on mechanisms involved in intracellular Ca^2+^ handling, as discussed below.

**Table 1:** **Influence of chronic testosterone withdrawal on myocardial contractility**

**Component**	**Model**	**Functional change**	**Duration of GDX (weeks)**	**Species**	**Reference**
LVDP	Langendorff	↔	2	Rat	[75]
		↔	9	Rat	[76]
		^a^↓	16	Rat	[74]
	Working heart	^b^↓	3	Rat	[77]
		^b^↓	10	Rat	[78]
LVEDP	Millar catheterization	↔	10–11	Mouse	[69]
	Langendorff	↔	2	Rat	[75]
	Working heart	^b^↓	3	Rat	[77]
		^b^↓	10	Rat	[78]
+dP/dT	Millar catheterization	↔	10–11	Mouse	[69]
	Langendorff	↔	9	Rat	[76]
		^a^↓	16	Rat	[74]
−dP/dT	Millar catheterization	↔	10–11	Mouse	[69]
	Langendorff	↔	9	Rat	[76]
		^a^↓	16	Rat	[74]
	Working heart	^b^↓	3	Rat	[77]
		^b^↓	10	Rat	[78]

^a^Effect of GDX is reversed by testosterone replacement. ^b^Functional change in the working heart model was only observed at high left atrial pressures.

#### Long-term influence of testosterone on cellular Ca^2+^ handling

The process by which cardiac excitation triggers a rise in intracellular Ca^2+^ and contraction is known as excitation-contraction coupling (reviewed by [80,81]). This process is initiated when Ca^2+^ enters the cell via L-type Ca^2+^ channels during phase 2 of the action potential (AP). This small influx of Ca^2+^ triggers the release of a much larger amount of Ca^2+^ through Ca^2+^ release channels (ryanodine receptors (RyRs)) in the sarcoplasmic reticulum (SR) in a process known as Ca^2+^-induced Ca^2+^ release [12,81]. Ca^2+^ is released from the SR in the form of discrete, subcellular units called Ca^2+^ sparks that fuse to form the Ca^2+^ transient [82]. Ca^2+^ then binds to contractile proteins, known as myofilaments, which results in sarcomere shortening and cardiac contraction. Relaxation occurs when Ca^2+^ is taken back up into the SR by the sarco/endoplasmic reticulum Ca^2+^-ATPase (SERCA), whose activity is regulated by the endogenous inhibitor, phospholamban (PLB). Some Ca^2+^ is also removed from the cell on a beat-to-beat basis, primarily by the bidirectional Na^+^/Ca^2+^ exchanger (NCX) that removes one Ca^2+^ in exchange for 3 Na^+^ [83]. The effects of testosterone on cardiac contractile function may arise through effects on components of the excitation-contraction coupling pathway at the level of the myocyte.

#### Testosterone and the cardiac action potential

As SR Ca^2+^ release and contraction are initiated by the cardiac AP, some investigators have explored the influence of testosterone on AP properties (Table 2). Chronic exposure to testosterone itself has no effect on resting membrane potential (RMP), AP amplitude, or AP duration at 50% repolarization (APD_50_) but actually prolongs APD_95_ in rat papillary muscle [84]. By contrast, more recent work in isolated mouse ventricular myocytes has shown that, while chronic DHT treatment has no effect on RMP, it does cause a marked abbreviation of both APD_50_ and APD_90_ [39]. One factor that might account for discrepancies between these two studies is the difference between chronic exposures to testosterone versus DHT. As reviewed in the ‘Biosynthesis of testosterone’ section, testosterone can be converted to estrogen by the enzyme aromatase whereas DHT cannot [33,36]. Therefore, estrogen may contribute to observed effects in studies where testosterone is used as the androgen receptor ligand [84]. Consistent with this idea, chronic exposure to estrogen has been shown to prolong APD in some models [57], in particular, when animals are in the estrus stage where estrogen levels peak [85-87]. Thus, apparent prolongation of APD by testosterone may be due to estradiol produced by aromatization. Certainly the DHT data [39] suggest that chronic exposure to androgens actually abbreviates APD.

**Table 2:** **Impact of chronic androgen treatment and GDX on resting and action potentials**

^**a**^ **Component**	**Treatment**	**Duration (weeks)**	**Preparation**	**Functional change**	**Species**	**Reference**
*Testosterone supplementation*						
RMP	^b^Testosterone	4	Ventricular muscle	↔	Rat	[84]
	^c^Dihydrotestosterone	13	Ventricular myocyte	↔	Mouse	[39]
AP amplitude	^b^Testosterone	4	Ventricular muscle	↔	Rat	[84]
APD50	^b^Testosterone	4	Ventricular muscle	↔	Rat	[84]
	^c^Dihydrotestosterone	13	Ventricular myocyte	↓	Mouse	[39]
APD90/95	^b^Testosterone	4	Ventricular muscle	↑	Rat	[84]
	^c^Dihydrotestosterone	13	Ventricular myocyte	↓	Mouse	[39]
*Gonadectomy* (*GDX*)						
RMP	GDX	4	Ventricular muscle	↔	Rat	[84]
		11–15	Ventricular myocyte	↔	Mouse	[39]
AP amplitude	GDX	4	Ventricular muscle	↔	Rat	[84]
APD50	GDX	4	Ventricular muscle	↑	Rat	[84]
		11–15	Ventricular myocyte	↑	Mouse	[39]
APD90/95	GDX	4	Ventricular muscle	↔	Rat	[84]
		11–15	Ventricular myocyte	↑	Mouse	[39]

^a^All experiments used paced tissues and myocytes from male animals only. ^b^Testosterone treatment with 5 mg/kg/day. ^c^Dihydrotestosterone treatment with a 7.5-mg pellet.

Information about the effects of testosterone on the cardiac AP also has been inferred from studies where animals were subjected to bilateral GDX through removal of the testes. The results of these investigations also are summarized in Table 2. It seems clear that GDX has no effect on either RMP or AP amplitude [39,84]. By contrast, there is general agreement that GDX prolongs APD_50_ [39,84] and APD_90/95_ ([39] but *c.f.* [84]) in both intact ventricular muscle and in isolated ventricular myocytes (Table 2). Taken together with the DHT work reviewed above, these observations suggest that chronic exposure to testosterone abbreviates the cardiac AP, and in its absence, APD is prolonged. The shape of the cardiac AP is inextricably linked to SR Ca^2+^ release and contraction [85]. Thus, the increase in APD seen following GDX may prolong SR Ca^2+^ release, increase the duration of contraction, and slow relaxation. Thus, prolongation of the AP may help explain the prolonged relaxation times seen in the hearts of GDX animals (Table 1).

#### The influence of testosterone on transarcolemmal ion fluxes

GDX may act to prolong the cardiac AP by modifying ionic currents to either attenuate repolarization or prolong depolarization. Therefore, a number of investigators have examined the influence of GDX on sarcolemmal proteins and currents, as shown in Table 3. Most studies have examined repolarizing K^+^ currents in rodent models 13 to 16 weeks after GDX (Table 3). There is general agreement that GDX has no effect on transient outward current (I_TO_), steady state K^+^ current (I_SS_) and inward rectifier K^+^ current (I_K1_), or on the expression of proteins or mRNA levels linked to these currents [38,74]. By contrast, GDX reduces the magnitude of the ultra-rapid delayed rectifier K^+^ current (I_Kur_) and decreases the expression of the corresponding Kv1.5 protein ([38] but *c.f.* [74]). This reduction in peak I_Kur_ could contribute to the prolongation of the APD in rodent models. In the rabbit model, where the slow delayed rectifier K^+^ current (I_Ks_) is the major repolarizing current [88], peak I_Ks_ also is slightly reduced by GDX [89]. These observations suggest that long-term testosterone withdrawal attenuates repolarizing currents, which may help explain the longer APs seen in cardiac muscle and myocytes from GDX animals.

**Table 3:** **Influence of gonadectomy on sarcolemmal proteins and currents**

**Component**	**Treatment**	**Duration (weeks)**	**Functional change**	**Species**	**Reference**
Ultra-rapid delayed rectifier K+ current (IKur)	^a^GDX	13	↓ peak I_Kur_	Mouse	[38,39]
		13	↓ Kv1.5 protein	Mouse	[38,39]
	GDX	16	↔ Kv1.5 protein	Rat	[74]
Transient outward K+ current (ITO)	GDX	13	↔ peak I_TO_	Mouse	[38]
		13	↔ Kv4.2/4.3 protein	Mouse	[38]
Steady state K+ current (ISS)	GDX	13	↔ peak I_SS_	Mouse	[38]
		13	↔ Kv1.2 protein	Mouse	[38]
Inward rectifier K+ current (IK1)	GDX	13	↔ peak I_K1_	Mouse	[38]
		13	↔ Kir2.1 protein	Mouse	[38]
		16	↓ Kir2.1 mRNA	Rat	[74]
		16	↔ Kir2.2 mRNA	Rat	[74]
		16	↔ Kir2.3 mRNA	Rat	[74]
Slow delayed rectifier K+ current (IKs)	GDX	8	↓ peak I_Ks_	Rabbit	[89]
L-type Ca2+ current (ICa-L)/ dihydropyridine receptor (DHPR)	GDX	9	↔ peak I_Ca-L_	Rabbit	[40]
	^b^GDX	2	↓ DHPR mRNA	Rat	[90]
		16	↓ DHPR mRNA	Rat	[91]
Na + -Ca2+ exchanger	GDX	10	↔ NCX activity	Rat	[96]
		9	↔ NCX activity	Rat	[76]
		2	↔ NCX protein	Rat	[75]
		10	↓ NCX protein	Rat	[96]
		10–11	↑ NCX protein	Mouse	[69]
	^b^GDX	2	↓ NCX mRNA	Rat	[90]
		16	↓ NCX mRNA	Rat	[91]

^a^Indicates that the effect of GDX was reversed by treatment with dihydrotestosterone. ^b^Indicates that the effect of GDX was reversed by treatment with testosterone.

Prolongation of the AP by GDX also could arise through changes in ionic currents that prolong depolarization. Whether Na^+^ currents are influenced by GDX has not yet been investigated. However, a few studies have investigated the effect of GDX on L-type Ca^2+^ current (I_Ca-L_), as shown in Table 3. Voltage-clamp studies show that GDX has no effect on peak I_Ca-L_, at least in the rabbit model [40]. By contrast, the density of 1,4-dihydropyridine (DHP) receptors (L-type Ca^2+^ channels) is markedly reduced by GDX in hearts from male rodents, and this effect is reversed by testosterone replacement [90,91]. This suggests that GDX may actually reduce Ca^2+^ influx in the heart. In support of this, peak L- and T-type Ca^2+^ currents are enhanced in neonatal rat cardiomyocytes chronically exposed to testosterone (24–30 h in culture), an effect blocked by the nuclear androgen receptor antagonist, flutamide [92,93]. Chronic exposure to DHT also increases peak I_Ca-L_ and increases the expression of Ca_v_1.2 (the pore-forming subunit of the L-channel) in cultured human ventricular myocytes [94]. These findings suggest that testosterone increases inward Ca^2+^ currents and that this effect is attenuated by GDX. Thus, Ca^2+^ influx may actually be inhibited by GDX, so enhanced Ca^2+^ influx does not account for the increase in APD observed in the GDX heart. On the other hand, Ca^2+^ influx via I_Ca-L_ is the primary trigger for SR Ca^2+^ release. Thus, effects of testosterone on I_Ca-L_ could have important effects on cardiac contractility as discussed in the next section.

Another important sarcolemmal protein that regulates intracellular Ca^2+^ levels is NCX. This exchanger primarily operates to remove one Ca^2+^ from the cell in exchange for three Na^+^ [95]. This generates an inward current that helps maintain the AP plateau [95] and could, in theory, help prolong APD in GDX. Whether NCX is modified by testosterone has been investigated in GDX rodents with biochemical and molecular approaches (Table 3). While several studies have shown that NCX activity and protein expression are unchanged 2 to 10 weeks after GDX [75,76,96], others report that NCX protein levels are reduced ([96] but *c.f.* [69]). There is also evidence that mRNA levels decline after both short-term (2 weeks) and long-term (16 weeks) GDX, and this effect is abolished by testosterone supplementation [90,91], as shown in Table 3. As NCX helps remove Ca^2+^ from the cell, a reduction in NCX in GDX hearts could slow relaxation, as observed in perfused hearts from GDX animals (Table 1). Still, at present, there is no consensus on the effect of GDX on NCX, and it is uncertain whether NCX helps prolong the APD.

#### Testosterone affects contractions and Ca^2+^ release in individual cardiomyocytes

Previous experimental studies have shown that chronic testosterone withdrawal attenuates cardiac contractility *in vivo* (the ‘The impact of testosterone on cardiac contractile function in animal models’ section). GDX also reduces peak contraction and slows relaxation in isolated perfused hearts, especially when hearts are working under high loads (the ‘The impact of testosterone on cardiac contractile function in animal models’ section; Table 1). These changes in cardiac contractile function could arise, in part, because the ability of individual ventricular myocytes to contract is modified by testosterone. For example, there is some evidence that cardiomyocyte contractions are larger in cells from male animals when compared to females (reviewed by [57]). Furthermore, chronic testosterone treatment (24 h in culture) enhances peak contractions, measured as unloaded cell shortening, in isolated rat cardiomyocytes [97]. Larger contractions are also observed in cells from female aromatase knockout mice, who have elevated testosterone levels along with low-estrogen levels [98]. Studies in myocytes from GDX rats also provide some support for these observations (Table 4). While one investigation showed that peak contractions are not affected by GDX [91], another found that peak responses are attenuated by testosterone deprivation [99]. On balance, these studies suggest that testosterone enhances peak contraction and testosterone deprivation may suppress contraction at the level of the cardiomyocyte.

**Table 4:** **Gonadectomy modifies cardiomyocyte Ca** ^**2+**^ **release and contraction**

**Parameter**	**Component**	**Functional change**	**[Ca** ^**2+**^ **] (mM)**	**Pacing rate (Hz)**	**Duration of GDX (weeks)**	**Species**	**Reference**
Cell shortening	Peak contraction	↔	1	0.5	16	Rat	[91]
		^a^↓	0.5–2	0.5	2	Rat	[99]
	Relaxation rate	^a^Slowed	1	0.5	16	Rat	[91]
			0.5–2	0.5	2	Rat	[99]
Intracellular Ca2+	Peak Ca^2+^ transient	↔	1	0.2	9	Rat	[76]
		^a^↓	0.5–2	0.5	2	Rat	[99]
	Ca^2+^ transient decay rate	^a^Slowed	0.5–2	0.5	2	Rat	[99]
			1	0.2	9	Rat	[76]

^a^Indicates that the effect of GDX was reversed by treatment with testosterone in studies by Curl et al. [99] and Golden et al. [91].

Other studies have investigated the influence of testosterone on the rate of relaxation of contraction. There is evidence that the rates of relaxation are faster in cardiomyocytes from male animals than in cells from females (reviewed by [57]). Likewise, chronic exposure to testosterone (24 h in culture) increases the rate of relaxation in individual rat myocytes [97], while testosterone withdrawal slows cardiomyocyte relaxation [91,99], as shown in Table 4. Female aromatase knockout mice (with high testosterone and low estrogen) also exhibit faster contractions than wild type controls [98]. These findings suggest that the slower − dP/dT reported in many studies of perfused GDX hearts ([74,77,78] but *c.f.* [69,76]) is attributable, at least in part, to prolongation of contraction at the cellular level.

Testosterone could modify cardiac contractile function by effects on the contractile proteins themselves. However, few studies have examined the impact of testosterone on myofilament proteins. One study has shown that testosterone deprivation has no effect on myofilament Ca^2+^ sensitivity but reduces maximal myofilament responses to Ca^2+^ in skinned rat ventricular muscle [96]. This decrease in maximal responsiveness to Ca^2+^ could contribute to the reduction in contractility observed in GDX hearts. Another study has examined myosin heavy chain (MHC) composition in sham-operated and GDX rat hearts [100]. They found that GDX causes a shift from the fast α-MHC isoform to the slower ß-MHC isoform, and this is reversed by testosterone replacement [100]. Predominance of the slower ß-MHC isoform could contribute to the slowing of relaxation in isolated cardiomyocytes and perfused hearts from GDX animals.

Others have investigated whether the influence of testosterone on cardiac contraction is mediated by changes in underlying Ca^2+^ transients (Table 4). There is some evidence that Ca^2+^ transients are larger and, in particular, faster in myocytes from males than females (reviewed by [57]). Female aromatase knockout mice (high-testosterone and low-estrogen levels) also exhibit larger and faster Ca^2+^ transients when compared to wild type [98]. Furthermore, although one study showed that Ca^2+^ transients are not affected by GDX [76], another reported that peak responses are attenuated by GDX [99]. As with myocyte contraction, there is general agreement (Table 4) that GDX slows the rate of decay of the Ca^2+^ transient in isolated cardiomyocytes [76,99]. These findings suggest that the smaller, slower contraction characteristic of GDX myocytes and hearts are attributable, at least in part, to changes in the underlying Ca^2+^ transients.

Interestingly, studies that report no change in peak contractions and Ca^2+^ transients after GDX [76,91] use external Ca^2+^ concentrations and pacing frequencies far below physiological for rats (e.g., 1 mM Ca^2+^; 0.2–0.5 Hz). By contrast, Curl et al. [99], who also used low-pacing rates (e.g., 0.5 Hz), used a range of external Ca^2+^ concentrations (e.g., 0.5–2 mM) and found that peak contractions and Ca^2+^ transients declined after GDX, especially at higher external Ca^2+^ concentrations (Table 4). These data suggest that the influence of GDX on peak cardiac contraction may become evident when cardiomyocytes are working under more physiological conditions. As the impact of GDX on contractility in perfused hearts is greatest when hearts are working at high loads [77,78], experiments that expose GDX myocytes to higher pacing frequencies could be informative.

In theory, testosterone may modulate other aspects of Ca^2+^ handling in the cardiomyocyte. For example, whether the rate of rise of the Ca^2+^ transient is modified by GDX has not yet been investigated. Changes in the Ca^2+^ transient rise time in GDX hearts could reflect de-synchrony of Ca^2+^ release mechanisms in the SR [101]. Furthermore, whether diastolic Ca^2+^ levels are modified by testosterone has not been examined. Higher diastolic Ca^2+^ levels would be expected if SR Ca^2+^ sequestration is slowed by GDX, as suggested by the longer Ca^2+^ transient decay rates seen in cells from GDX animals [76,99]. The next section reviews studies that have investigated the influence of chronic testosterone withdrawal on specific intracellular Ca^2+^-handling mechanisms in the heart.

#### Testosterone targets SR Ca^2+^-handling mechanisms in the cardiomyocyte

The SR plays a crucial role in Ca^2+^ release and reuptake within the cardiomyocyte, so a number of investigators have examined the influence of testosterone on SR Ca^2+^ release mechanisms (Table 5). As with most studies, the influence of testosterone on processes involved in SR Ca^2+^ handling has largely been inferred from studies done on animals subjected to GDX. Although there is no indication that RyR protein levels are affected by GDX [75], RyR-mediated ^45^Ca^2+^ flux is actually reduced following GDX in the rat model [76]. In support of this observation, chronic exposure to testosterone (24–30 h of culture) increases the amplitude of subcellular SR Ca^2+^ release units known as Ca^2+^ sparks [92]. Furthermore, Ca^2+^ sparks are larger in cardiomyocytes from male animals when compared to females [102,103]. Taken together, these findings suggest that testosterone enhances SR Ca^2+^ release by increasing the magnitude of individual Ca^2+^ sparks and that chronic testosterone withdrawal suppresses this process. If SR Ca^2+^ release events are reduced by GDX, this could account for the reduction in peak contraction observed in both intact hearts and cardiomyocytes from GDX animals. Additional studies that explore the role of testosterone in regulating Ca^2+^ sparks via signaling pathways such as those mediated by PKA and Ca^2+^ calmodulin-dependent kinase II (CaMKII) [102,104,105] would be of interest.

**Table 5:** **Effect of gonadectomy on Ca** ^**2+**^ **handling by the sarcoplasmic reticulum**

**Component**	**Functional change**	**Duration of GDX (weeks)**	**Species**	**Reference**
SR Ca2+ release	↔ RyR2 protein	2	Rat	[75]
	^a^ ↓ RyR-mediated ^45^Ca^2+^ flux	9	Rat	[76]
SR Ca2ccps stores	↔ Calsequestrin protein	10-11	Mouse	[69]
	↔ Calreticulin protein	10-11	Mouse	[69]
	^a^↓ SR Ca^2+^ content	9	Rat	[76]
SERCA2a	↔ SERCA2a protein	2	Rat	[75]
		10	Rat	[96]
		10-11	Mouse	[69]
	↔ SERCA activity	10	Rat	[76]
	^a^↓ SERCA activity	9	Rat	[96]
PLB	↔ PLB protein	2	Rat	[75]
		10	Rat	[96]
		9	Rat	[76]
		10-11	Mouse	[69]
	^a^↓ PLB Thr^17^ phosphorylation	10-11	Mouse	[69]
		10	Rat	[96]
	↓ PLB Ser^16^ phosphorylation	10-11	Mouse	[69]
	↔ PLB Ser^16^ phosphorylation	10	Rat	[96]

^a^Indicates that the effect of GDX was reversed by treatment with testosterone in studies by Tsang et al. [76] and Witayavanitkul et al. [96].

Most investigations of the influence of testosterone on SR function have focussed on its effects on SR Ca^2+^ content and SR Ca^2+^ reuptake mechanisms. Studies have shown that levels of the SR Ca^2+^-binding proteins calsequestrin and calreticulin are not affected by GDX [69]. However, SR Ca^2+^ content is reduced in myocytes from GDX animals when compared to sham-operated controls [76]. The mechanism responsible for the reduction in SR Ca^2+^ content in the setting of chronic testosterone withdrawal has been investigated. Table 5 shows that the expression of the cardiac SR Ca^2+^ ATPase protein, SERCA2a, is not affected by GDX [69,75,96]. Thus, there is no evidence that a reduction in the expression of SERCA2a can account for the decrease in SR Ca^2+^ content in GDX hearts.

Other investigators have examined the expression of the endogenous SERCA2a inhibitor, PLB, after chronic testosterone withdrawal (Table 5). There is good agreement that PLB protein expression is similar in sham-operated and GDX hearts [69,75,76,96]. However, the regulation of PLB by key signaling pathways is modified by GDX. It is well known that PLB is regulated by phosphorylation through both PKA and CaMKII pathways [106]. As shown in Table 5, there is strong evidence that phosphorylation of PLB at the CaMKII site (Thr^17^) is reduced by GDX [69,96]. There is also evidence that PLB phosphorylation at the PKA site (Ser^16^) is reduced by GDX [69], although this is not seen in all studies [96]. As phosphorylation of PLB at both the PKA and CaMKII sites increases the activity of SERCA2a [106], a reduction in phosphorylation at these sites would be expected to reduce SERCA2a activity. This agrees with reports that SERCA activity is reduced by GDX ([96] but *c.f.* [76]). This reduction in the rate of SR Ca^2+^ may explain the slower Ca^2+^ transient decay [76,99] and reduction in SR Ca^2+^ content [76] characteristic of cardiomyocytes from GDX animals.

Although information is limited, there is evidence that the cAMP/PKA and CaMKII pathways are modulated by androgens. It is known that cAMP levels are higher in cardiomyocytes from young adult male mice when compared to females [102]. While this is due, at least in part, to increased expression of phosphodiesterase 4B in female cells [102], testosterone also may play a role. In support of this, testosterone has been shown to inhibit phosphodiesterase and increase cAMP levels in rat atria and ventricles [107]. Thus, cAMP would be expected to fall in animals subjected to chronic GDX. Testosterone-associated changes in the CaMKII pathway also are likely to occur in the setting of GDX, as CaMKII is activated by intracellular Ca^2+^, which is reduced by GDX (Table 4). Additional studies that explore the impact of androgens on pathways involved in post-translational modifications of SR Ca^2+^ release mechanisms in the heart would be of interest.

### Acute effects of testosterone on cardiac Ca^2+^-handling mechanisms

Although most studies have explored the effects of chronic testosterone exposure and/or deprivation on the heart, others have examined acute (nongenomic) effects of testosterone in cardiac muscle and isolated cardiomyocytes. It is well established that physiological testosterone levels fluctuate between 10 and 35 nM in adult men [108], and similar levels are seen in male rodents [109]. However, as outlined below, experimental studies of acute testosterone application have typically used supra-physiological concentrations to assess actions on cardiac Ca^2+^-handling mechanisms.

There is evidence that testosterone can acutely affect the cardiac AP, at least when higher concentrations of androgens are used. Acute application of testosterone has no effect on RMP but prolongs APD in guinea pig papillary muscle preparations when high concentrations are used (e.g., 1,000 μM) [110]. On the other hand, acute application of lower concentrations of testosterone (e.g., 100 nM) actually shortens APD in guinea pig ventricular myocytes [111]. Furthermore, lower concentrations of testosterone also can acutely affect ionic currents in isolated cardiomyocytes. For example, Michels et al. [93] reported that 10 μM testosterone reduces peak T-type Ca^2+^ current in neonatal rat cardiomyocytes. Furthermore, even lower concentrations of testosterone (100 nM) inhibit peak I_Ca-L_ in ventricular myocytes isolated from neonatal rats [92] and adult guinea pigs [111]. Superfusion of isolated rabbit ventricular myocytes with 3 to 10 nM concentrations of the potent androgen DHT increases the magnitude of I_K1_ [112] and application of 100 nM testosterone enhances I_Ks_ in guinea pig ventricular myocytes [111]. These studies demonstrate that testosterone and its analogs can acutely modify the cardiac AP and underlying ionic currents, although whether these effects are relevant *in vivo* is unclear because most studies used supra-physiological concentrations of drug.

Others have examined effects of acute testosterone application on cardiomyocyte Ca^2+^ handling directly. One group has shown that acute application of testosterone (100 nM) elicits voltage-dependent oscillations in intracellular Ca^2+^, along with IP_3_-receptor-mediated Ca^2+^ release from SR stores in neonatal rat ventricular myocytes [53]. By contrast, another study demonstrated that acute application of 1 μM testosterone had no effect on either the amplitudes or time courses of contractions in ventricular myocytes isolated from adult female rats [113]. This latter study also showed that 1 μM testosterone did not affect the amplitude or time course of the Ca^2+^ transient [113]. Whether differences between neonatal and adult ventricular myocytes account for these differing results is not yet clear. Importantly, the Beesley et al. [113] study showed that even high concentrations of testosterone have no acute effects on Ca^2+^ homeostasis or contractions in cells from females. As most studies of the effects of testosterone on the heart have used cells from males, it is possible that there are male-female differences in responses to testosterone, and additional studies to address this issue are needed. These acute effects of androgens on the heart are seen only at high concentrations of steroid, so the physiological relevance of these findings is uncertain. Additional studies that explore the impact of more physiological concentrations on cardiac Ca^2+^-handling mechanisms are warranted.

## Conclusions

**Figure 2:**
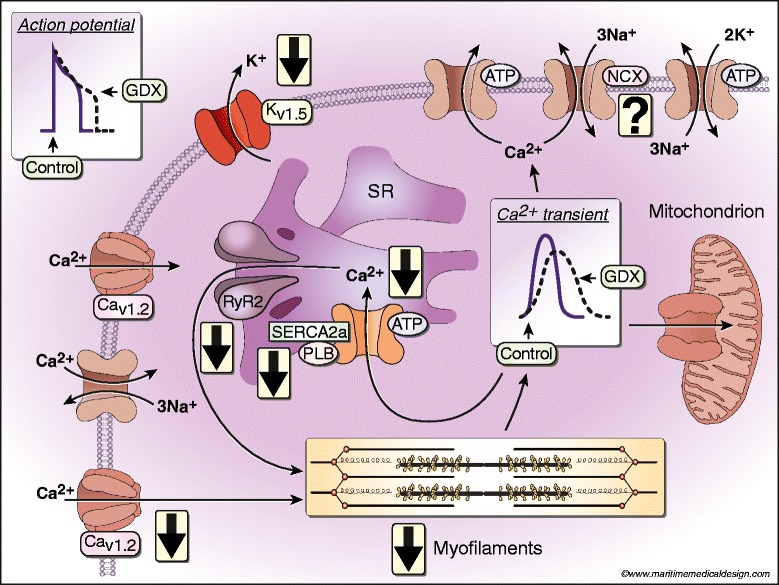
Impact of GDX on intracellular Ca^2+^-handling mechanisms in ventricular myocytes isolated from rodent hearts. APD is prolonged by GDX, due to a decrease in repolarizing K^+^ currents (I_Kur_) and a reduction in the expression of Kv1.5. Reduced Ca^2+^ influx along with smaller Ca^2+^ sparks attenuates SR Ca^2+^ release. Ca^2+^ transient decay is slowed by longer APs and slower SR Ca^2+^ uptake mediated by a decrease in phosphorylation of PLB by CaMKII (and possibly PKA). Peak contractions are attenuated through smaller peak Ca^2+^ transients and a decrease in maximal myofilament responsiveness to Ca^2+^. Contractions are slowed because SR Ca^2+^ uptake is reduced and the slower β-MHC isoform predominates. Whether NCX activity or expression is affected by GDX is not yet clear.

The evidence reviewed here suggests that chronic testosterone withdrawal influences cardiac Ca^2+^-handling mechanisms in ventricular myocytes, as illustrated in Figure 2. APD is prolonged in the absence of testosterone, an effect mediated by a decrease in magnitude of the repolarizing K^+^ current, I_Kur_, at least in rodent models. This reduction in I_Kur_ is secondary to a decrease in the expression of Kv1.5. Ca^2+^ transients also are smaller and slower in ventricular myocytes from GDX animals when compared to sham-operated controls, especially when cells are paced at physiological rates. The decrease in SR Ca^2+^ release arises as a consequence of changes in several components of the excitation-contraction coupling pathway. First, GDX reduces the density of L-type Ca^2+^ channels, so Ca^2+^ influx is reduced and there is less Ca^2+^ available to trigger SR Ca^2+^ release. Second, the amount of SR Ca^2+^ available for release is reduced by GDX, and the magnitude of Ca^2+^ sparks may decline. The decay of the Ca^2+^ transient is slowed as a consequence of a decrease in the rate of SR Ca^2+^ uptake along with prolongation of the APD. The decline in SR Ca^2+^ uptake arises through a reduction in phosphorylation of PLB by CaMKII and possibly also by PKA. Contractions are attenuated in GDX myocytes due to a decrease in the magnitude of the Ca^2+^ transient along with a reduction in the maximal myofilament responsiveness to Ca^2+^. Relaxation is slowed due to slower Ca^2+^ transient decay along with a shift from the fast α-MHC isoform to the slower β-MHC isoform. These findings demonstrate that GDX influences critical mechanisms involved in Ca^2+^ homeostasis and suggest that testosterone modulates myocardial function, at least in part, by effects on individual ventricular myocytes in rodent models. Additional experiments that explore the impact of testosterone on these mechanisms in cardiomyocytes from larger mammals including humans are needed and the key signaling pathways involved should be identified.

The idea that testosterone regulates the cardiac action potential and Ca^2+^ homeostasis at the level of the individual heart cell has a number of important clinical implications. For example, men have faster rates of repolarization than women [114], and castrated men have prolonged repolarization while the reverse is seen in women with abnormally high levels of testosterone [115]. This is consistent with evidence that GDX increases APD in individual myocytes in animal models. This may be clinically important as prolongation of the AP can increase the probability of early after depolarizations, which can trigger arrhythmias such as torsades des pointes [116,117]. Furthermore, it is well known that levels of testosterone decline with age, at the same time as the incidence of cardiovascular disease rises. Modifications in myocardial Ca^2+^ handling and contraction linked to falling testosterone levels in older adults are likely to interact with diseases in the aging heart. For example, the observation that contractions and Ca^2+^ transients decline in low-testosterone states may promote heart failure with reduced ejection fraction [118]. Intracellular Ca^2+^ dysregulation also is implicated in the pathogenesis of diseases such as myocardial ischemia and arrhythmias [119], where a decrease in testosterone may influence disease expression. Improved understanding of the cellular mechanisms involved in the effects of testosterone on the heart may reveal mechanisms involved in the increase in susceptibility to cardiovascular diseases in aging and may ultimately help identify new targets for intervention in the treatment of these diseases in both men and women.

## Abbreviations

AP: Action potential
APD_50_: AP duration at 50% repolarization
CaMKII: Ca^2+^ calmodulin-dependent kinase II
CYP: Cytochrome P450
DHP: 1,4-dihydropyridine
DHEA: Dehydroepiandrosterone
DHT: Dihydrotestosterone
GDX: Gonadectomy
GnRH: Gonadotropin-releasing hormone
HSD3B: 3β-hydroxysteroid dehydrogenase
HSD17B3: 17β-hydroxysteroid dehydrogenase 3
I_K1_: Inward rectifier K^+^ current
LVDP: Left ventricular developed pressure
LVEDP: Left ventricular end-diastolic pressure
I_Ca-L_: L-type Ca^2+^ current
LH: Luteinizing hormone
MHC: Myosin heavy chain
PLB: Phospholamban
PKA: Protein kinase A
+dP/dT: Rate of left ventricular pressure rise
−dP/dT: Rate of left ventricular pressure decay
RMP: Resting membrane potential
RyR: Ryanodine receptor
SERCA: Sarco/endoplasmic reticulum Ca^2+^-ATPase
SR: Sarcoplasmic reticulum
I_Ks_: Slow delayed rectifier K^+^ current
NCX: Na^+^/Ca^2+^ exchanger
I_SS_: Steady state K^+^ current
StAR: Steroidogenic acute regulatory
I_TO_: Transient outward current
I_Kur_: Ultra-rapid delayed rectifier K^+^ current
